# Supplementary material for: Do you have COVID-19? How to increase the use of diagnostic and contact tracing apps
Source: PLoS One. 2021 Jul 29;16(7):e0253490. doi: 10.1371/journal.pone.0253490 (PMC8321141; doi:10.1371/journal.pone.0253490)
Supplement: S1 Fig — The figure shows a couple of examples of the ads used for recruitment. S2 Fig shows the different combinations of pictures used to construct these ads. These ads were designed by the project team and the IDB communications team. (PDF) [file pone.0253490.s001.pdf]

Supporting information

Do you have COVID-19? How to increase the use of diagnostic and contact tracing apps

Deborah Martínez, Cristina Parilli, Ana M. Rojas, Carlos Scartascini, Alberto Simpser

S1 Fig. Facebook Ads - Recruitment. The figure shows a couple of examples of the ads used for recruitment. S2 Fig shows the different combinations of pictures used to construct these ads. These ads were designed by the project team and the IDB communications team.

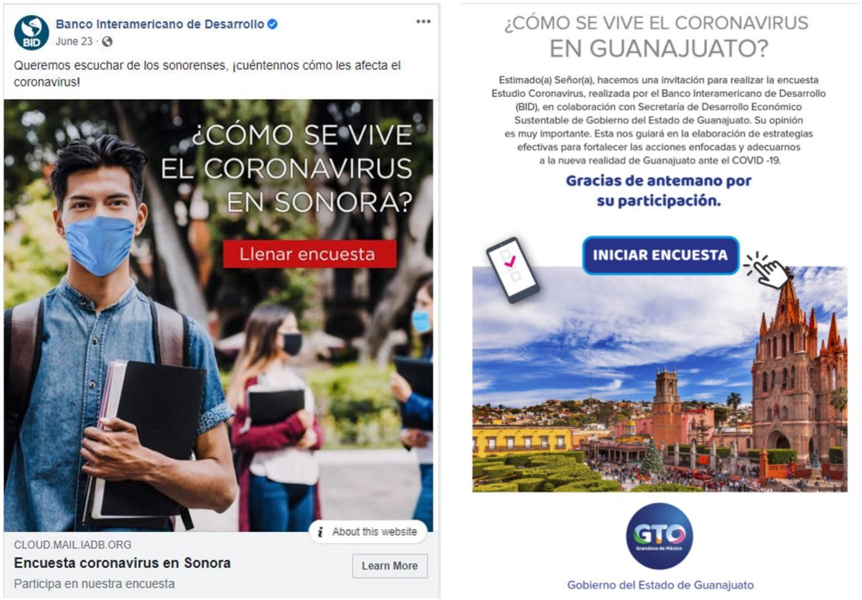

Translation of left ad	Translation of right ad
Inter-American Development Bank	How do you live with Coronavirus in Guanajuato?
We want to hear from Sonorans. Tell us how the coronavirus is affecting you!	Dear Madam[/Sir], we invite you to fill out the Coronavirus Study survey, carried out by the Inter-American Development Bank (IDB), in collaboration with the Secretariat for Sustainable Economic Development of the Government of the State of Guanajuato. Your opinion is very important. it will guide us in the development of effective strategies to strengthen focused actions and adapt to the new reality of Guanajuato in the face of COVID-19.
How is the coronavirus experienced in Sonora?	Thanks in advance for your participation.
Fill out the survey	Start survey
Sonora virus survey	Guanajuato state government
Take part in our survey	
