# Supplementary material for: Do you have COVID-19? How to increase the use of diagnostic and contact tracing apps
Source: PLoS One. 2021 Jul 29;16(7):e0253490. doi: 10.1371/journal.pone.0253490 (PMC8321141; doi:10.1371/journal.pone.0253490)

S2 Fig. Facebook Ads - Set of pictures for the ads. The figure shows the different pictures that were used to construct the set of ads used for recruitment.These ads were designed by the project team and the IDB communications team.

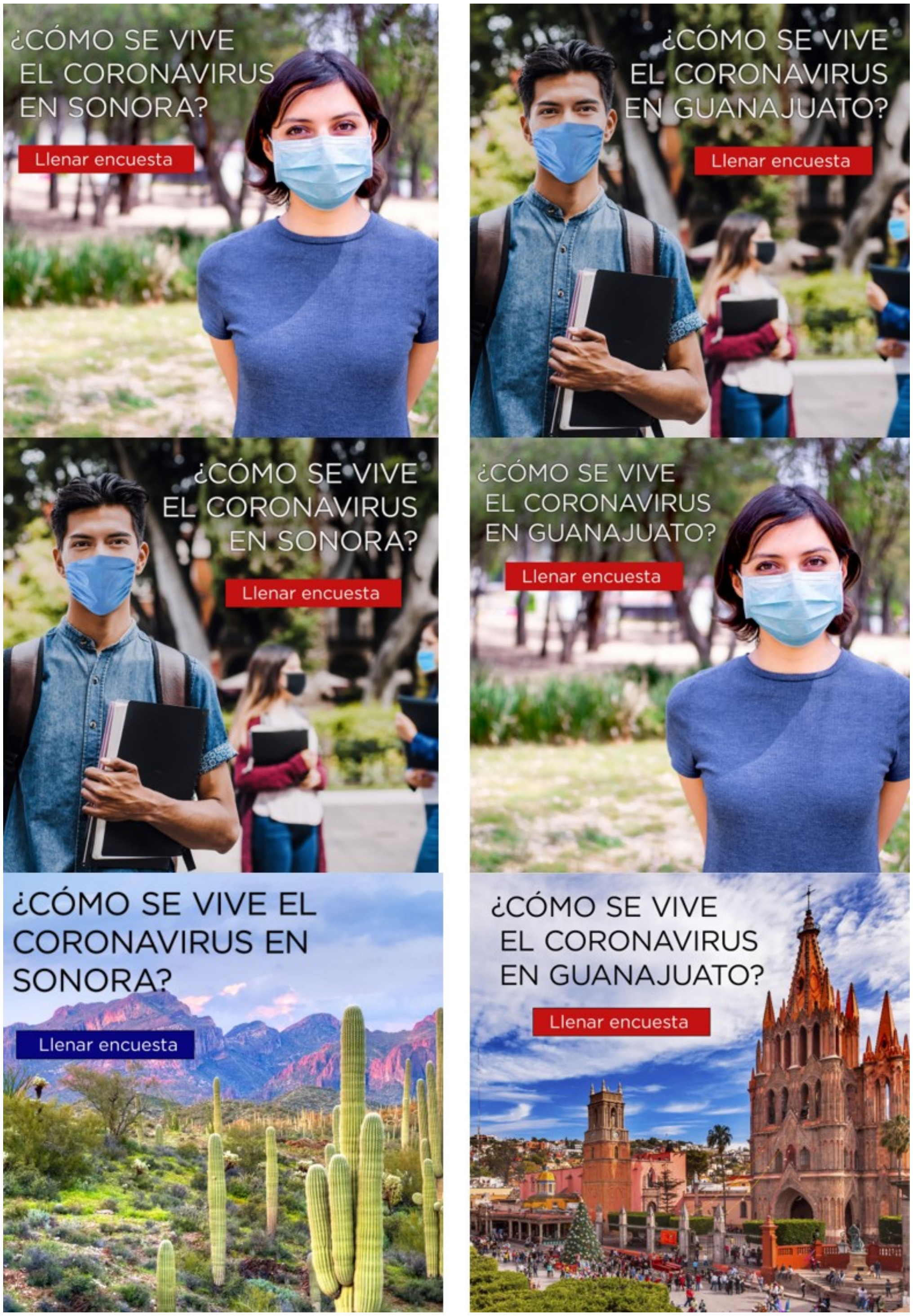

Supplement: S2 Fig — The figure shows the different pictures that were used to construct the set of ads used for recruitment.These ads were designed by the project team and the IDB communications team. (PDF) [file pone.0253490.s002.pdf]
