# Supplementary material for: Do you have COVID-19? How to increase the use of diagnostic and contact tracing apps
Source: PLoS One. 2021 Jul 29;16(7):e0253490. doi: 10.1371/journal.pone.0253490 (PMC8321141; doi:10.1371/journal.pone.0253490)

S3 Fig. Distribution of responses - Control group. This figure shows the distribution of responses to the questions regarding the download of the apps.

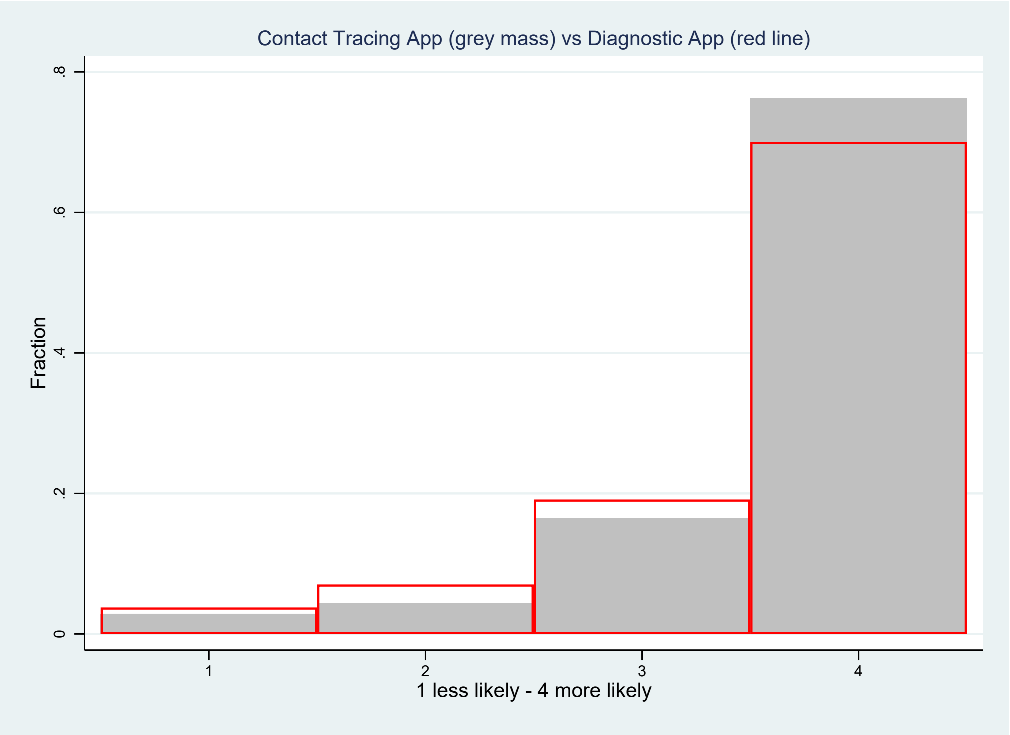

Supplement: S3 Fig — This figure shows the distribution of responses to the questions regarding the download of the apps. (PDF) [file pone.0253490.s003.pdf]
