# Supplementary material for: Do you have COVID-19? How to increase the use of diagnostic and contact tracing apps
Source: PLoS One. 2021 Jul 29;16(7):e0253490. doi: 10.1371/journal.pone.0253490 (PMC8321141; doi:10.1371/journal.pone.0253490)
Supplement: S1 Table — The Table shows the regression coefficients corresponding to an ordered logit regression keeping the dependent variables in their original categorical values. (PDF) [file pone.0253490.s004.pdf]

S1 Table. Willingness to download the app - Ordered Logistic Regression. The Table shows the regression coefficients corresponding to an ordered logit regression keeping the dependent variables in their original categorical values.

	Tracing App			Diagnostic App		
	(1)	(2)	(3)	(4)	(5)	(6)
T1 (Facebook)	-0.067	-0.083*	-0.077*	-0.084**	-0.096**	-0.096**
	(0.043)	(0.046)	(0.046)	(0.040)	(0.042)	(0.042)
T2 (GovOnlServ)	0.053	0.045	0.049	0.085**	0.090**	0.092**
	(0.044)	(0.046)	(0.046)	(0.040)	(0.042)	(0.043)
T3 (DataPrivacy)	-0.341***	-0.369***	-0.363***	-0.352***	-0.377***	-0.375***
	(0.042)	(0.045)	(0.045)	(0.040)	(0.042)	(0.042)
Observations	22,776	21,251	21,193	22,724	21,194	21,137
Controls	No	Yes	Yes	No	Yes	Yes
Fixed Effects	No	No	State	No	No	State
T1=T2=T3	0.000	0.000	0.000	0.000	0.000	0.000
T1=T2	0.005	0.005	0.006	0.000	0.000	0.000
T1=T3	0.000	0.000	0.000	0.000	0.000	0.000
T2=T3	0.000	0.000	0.000	0.000	0.000	0.000

Notes: Each row shows the regression coefficients and the standard error in parenthesis corresponding to an ordered logit regression. Dependent variables take the values 1 (definitely would not) to 4 (definitely would) according to the willingness of the respondent to download each one of the apps. Survey questions used for the construction of the dependent variables available in S1 Appendix. Standard errors are robust. *** $p<0.01$, ** $p<0.05$, * $p<0.1$. Controls include: sex, age, education, exposed to Covid, death to Covid, older than 65 at home, belief about infection probability, belief about hospitalization probability, attends party, visits family, risk inside evaluation, and others practice social distancing. Survey questions used for the construction of the control variables available in S1 Appendix. *Source:* Authors' calculations.
