# Supplementary material for: Do you have COVID-19? How to increase the use of diagnostic and contact tracing apps
Source: PLoS One. 2021 Jul 29;16(7):e0253490. doi: 10.1371/journal.pone.0253490 (PMC8321141; doi:10.1371/journal.pone.0253490)
Supplement: S2 Table — This table shows descriptive statistics and balance among treatment assignment for each observable characteristic contained in the survey, for the Sonora Sample. (PDF) [file pone.0253490.s005.pdf]

S2 Table. Balance Table - Sonora Sample. This table shows descriptive statistics and balance among treatment assignment for each observable characteristic contained in the survey, for the Sonora Sample.

	Control	Difference w.r.t. control				Observations
**Variable**	(av. & s.e.)	T1	T2	T3	T4	
	(1)	(2)	(3)	(4)	(5)	(6)
Age	1.317	0.066	0.067	0.054	0.024	976
	(0.035)	(0.051)	(0.052)	(0.050)	(0.050)	
1.Younger 25	0.271	-0.025	-0.017	0.002	0.021	976
	(0.032)	(0.044)	(0.045)	(0.044)	(0.046)	
1.Older 55	0.080	0.003	0.011	0.017	-0.016	976
	(0.019)	(0.028)	(0.029)	(0.028)	(0.027)	
1.Female	0.915	0.017	-0.001	0.016	0.031	981
	(0.020)	(0.027)	(0.029)	(0.026)	(0.026)	
Education (group)	2.613	0.055	0.029	0.033	0.052	976
	(0.043)	(0.060)	(0.061)	(0.059)	(0.059)	
1.College	0.678	0.043	0.011	0.029	0.030	976
	(0.033)	(0.047)	(0.047)	(0.046)	(0.047)	
1.Exposed Covid	0.900	0.006	-0.014	0.021	0.035	975
	(0.021)	(0.030)	(0.032)	(0.028)	(0.028)	
1.Death Covid	0.824	0.024	-0.024	0.005	-0.014	976
	(0.027)	(0.038)	(0.040)	(0.037)	(0.040)	
1.Older 65 at home	0.216	-0.037	0.020	-0.050	0.103**	977
	(0.029)	(0.040)	(0.043)	(0.039)	(0.045)	
Pr(Infection)	47.658	-1.264	1.729	-0.093	-1.478	972
	(2.035)	(2.863)	(2.867)	(2.740)	(2.925)	
Pr(Hospital)	44.919	-4.998**	-1.427	-2.570	-1.078	971
	(1.594)	(2.216)	(2.385)	(2.248)	(2.351)	
1.Attend Party	0.275	-0.041	-0.061	-0.056	-0.086*	979
	(0.032)	(0.044)	(0.044)	(0.042)	(0.043)	
1.Visit	0.570	-0.015	0.018	-0.030	-0.016	979
	(0.035)	(0.050)	(0.050)	(0.049)	(0.051)	
1.Risky Inside	0.695	-0.002	-0.048	0.005	0.063	982
	(0.033)	(0.047)	(0.048)	(0.045)	(0.045)	
1.Social Distance	0.337	0.129***	0.064	0.101*	0.020	979
	(0.034)	(0.049)	(0.049)	(0.048)	(0.049)	

Notes: Each row shows statistics for a different observable variable we have in the Sonora Sample. Survey questions that serve the basis for the variables here, are available in S1 Appendix. Column [1] shows the sample average and the standard deviation in parentheses for the control group. Columns [2]-[4] show the regression coefficient and the standard error in parentheses corresponding to an OLS regression. Column [5] shows the sample size for each regression. Standard errors are robust. *** p<0.01, ** p<0.05, * p<0.1. Variables *Age* and *Education* are tabulated according to ranges; as such they are categorical, with a higher category number referring to an older age and more years of education, respectively. 1.x refers to dummy variables.
Source: Authors' calculations.
