# Supplementary material for: Do you have COVID-19? How to increase the use of diagnostic and contact tracing apps
Source: PLoS One. 2021 Jul 29;16(7):e0253490. doi: 10.1371/journal.pone.0253490 (PMC8321141; doi:10.1371/journal.pone.0253490)
Supplement: S3 Table — This table presents the Average Treatment Effect for the Sonora Sample. (PDF) [file pone.0253490.s006.pdf]

**S3 Table. Willingness to download the app - Sonora Sample.** This table presents the Average Treatment Effect for the Sonora Sample.

| | Tracing App | | | Diagnostic App | | |
|---|---|---|---|---|---|---|
| | (1) | (2) | (3) | (4) | (5) | (6) |
| T1 (Facebook) | 0.009 | 0.019 | 0.012 | 0.008 | 0.017 | 0.006 |
| | (0.018) | (0.018) | (0.019) | (0.023) | (0.024) | (0.026) |
| T2 (GovOnlServ) | -0.025 | -0.020 | -0.021 | 0.018 | 0.024 | 0.021 |
| | (0.022) | (0.023) | (0.024) | (0.023) | (0.024) | (0.025) |
| T3 (DataPrivacy Orig) | -0.045** | -0.022 | -0.030 | -0.057** | -0.034 | -0.045 |
| | (0.023) | (0.022) | (0.023) | (0.028) | (0.028) | (0.029) |
| T4 (DataPrivacy Rev) | -0.037 | -0.035 | -0.042 | -0.056* | -0.058* | -0.069** |
| | (0.023) | (0.024) | (0.025) | (0.029) | (0.031) | (0.033) |
| Constant | 0.965*** | 1.011*** | 1.064*** | 0.939*** | 0.962*** | 0.780*** |
| | (0.013) | (0.060) | (0.062) | (0.017) | (0.068) | (0.166) |
| Observations | 965 | 916 | 915 | 968 | 919 | 918 |
| R-squared | 0.008 | 0.022 | 0.046 | 0.014 | 0.020 | 0.049 |
| Controls | No | Yes | Yes | No | Yes | Yes |
| Fixed Effects | No | No | State | No | No | State |
| T1=T2=T3=T4 | 0.040 | 0.037 | 0.071 | 0.005 | 0.012 | 0.009 |
| T1=T2 | 0.110 | 0.076 | 0.149 | 0.671 | 0.789 | 0.528 |
| T1=T3 | 0.015 | 0.043 | 0.053 | 0.018 | 0.055 | 0.074 |
| T1=T4 | 0.043 | 0.018 | 0.030 | 0.026 | 0.013 | 0.021 |
| T2=T3 | 0.435 | 0.952 | 0.738 | 0.005 | 0.031 | 0.017 |
| T2=T4 | 0.654 | 0.583 | 0.484 | 0.009 | 0.006 | 0.005 |
| T3=T4 | 0.755 | 0.605 | 0.688 | 0.983 | 0.465 | 0.497 |

*Notes:* Each row shows the regression coefficients and the standard error in parenthesis corresponding to an OLS regression. Dependent variables take the value 0-1 according to the willingness of the respondent to download each application. Survey questions used for the construction of the dependent variables are available in S1 Appendix. Standard errors are robust. *** p<0.01, ** p<0.05, * p<0.1. Controls include: sex, age, education, exposed to Covid, death to Covid, older than 65 at home, belief about infection probability, belief about hospitalization probability, attends party, visits family, risk inside evaluation, and others practice social distancing. Survey questions used for the construction of the control variables available in S1 Appendix.
*Source:* Authors' calculations.
