# Supplementary material for: Do you have COVID-19? How to increase the use of diagnostic and contact tracing apps
Source: PLoS One. 2021 Jul 29;16(7):e0253490. doi: 10.1371/journal.pone.0253490 (PMC8321141; doi:10.1371/journal.pone.0253490)
Supplement: S1 Appendix — This document presents all the questions (in Spanish—original language of the survey—and English) used to construct dependent, treatment, as well as control variables. (PDF) [file pone.0253490.s007.pdf]

S1 Appendix. Survey questions. This document presents all the questions (in Spanish - original language of the survey - and English) used to construct dependent and treatment variables, and covariates.

Dependent Variables

**Variable**	**Dummy**	**Discrete coding**	**Original Survey Question (Spanish)**	**Translated Survey Question**
			Si hubiera una aplicación móvil del gobierno federal para tu teléfono que te permitiera saber si tienes algún síntoma de coronavirus y te dijera qué hacer, sin costo y sin consumir datos, ¿la instalarías en tu teléfono?	If a federal government app were available for your smartphone that could help you to identify coronavirus symptoms, and inform you what to do, at no cost, and with no data usage, would you download it to your phone?
Diagnostic application	1	4	Seguro sí	Definitely yes
	1	3	Creo que sí	I think so
	0	2	Creo que no	I don't think so
	0	1	Seguro no	Definitely not
	.		No sé / prefiero no responder	I don't know / I prefer not to answer
			Si además de lo anterior, esa aplicación también te alertara si estuviste en contacto por más de 15 minutos con una persona infectada de coronavirus, y les notificara a las personas que estuvieron en contacto cercano contigo, sin identificar ningún nombre, ni el tuyo ni el de las otras personas, ¿la instalarías en tu teléfono?	If, in addition to the previously-described features, the app could also alert you if you had been in contact for more than 15 minutes with an infected person, and it notified the people who were near you if you became infected, without identifying personal information (yours or others'), would you download the app?
Contact Tracing application	1	4	Seguro sí	Definitely yes
	1	3	Creo que sí	I think so
	0	2	Creo que no	I don't think so
	0	1	Seguro no	Definitely not
	.		No sé / prefiero no responder	I don't know / I prefer not to answer

Control Variables			
**Variable**	**Coding**	**Original Survey Question (Spanish)**	**Translated Survey Question**
Age (group)		¿Cuál es tu edad?	How old are you?
	1	18-24	18-25
	1	25-39	25-40
	2	40-55	40-56
	2	55-64	55-65
	3	65+	65+
	.	No sé / prefiero no responder	I don't know / I prefer not to answer
1.Female		¿Cuál es tu género?	What is your gender?
	1	Femenino	Female
	0	Masculino	Male
	0	Otro	Other
	.	No sé / prefiero no responder	I don't know / I prefer not to answer
Education (group)		¿Cuál fue el último nivel educativo que completaste?	What was the highest level of education you completed?
	0	No fui a la escuela	I did not go to school
	1	Primaria	Primary
	1	Secundaria	Secondary
	2	Preparatoria	High School
	3	Superior o universitaria	Higher or university
	3	Maestría u otro nivel más avanzado	Master's degreer another more advanced level
	.	No sé / prefiero no responder	I don't know / I prefer not to answer
1. Exposed COVID-19		¿Tú o algún amigo, familiar o colega tuyo han tenido Coronavirus?	Have you or a friend, relative or colleague of yours had Coronavirus?
	1	Sí	Yes
	0	No	No
	.	No sé / prefiero no responder	I don't know / I prefer not to answer
1. Death COVID-19		¿Conoces a alguien que haya muerto por Coronavirus?	Do you know someone who has died from Coronavirus?
	1	Sí	Yes
	0	No	No
	.	No sé / prefiero no responder	I don't know / I prefer not to answer

Control Variables			
**Variable**	**Coding**	**Original Survey Question (Spanish)**	**Translated Survey Question**
1. Older 65 Home		Incluyéndote a ti, ¿en este momento vive en tu hogar algún adulto mayor de 65 años?	Including you, is there an adult over 65 living in your household at this time?
	1	Sí	Yes
	0	No	No
	.	No sé / prefiero no responder	I don't know / I prefer not to answer
Prob. Infection		En tu opinión, ¿qué tan probable es que tú te contagies de Coronavirus en los siguientes 6 meses?	In your opinion, how likely is it that you will get Coronavirus in the next 6 months?
	1-100	Barra deslizante: variable continua	Sliding bar: continuous variable
Prob. Hospital		En tu opinión, si una persona de tu edad se contagia de Coronavirus, ¿qué tan probable es que termine hospitalizado/a?	In your opinion, if a person your age is infected with Coronavirus, how likely is it that they will end up hospitalized?
	1-100	Barra deslizante: variable continua	Sliding bar: continuous variable
1. Attend Party		En los últimos 7 días, ¿tú o alguien en tu hogar realizaron alguna de las siguientes actividades?	In the last 7 days, did you or someone in your household perform any of the following activities?
		Visitar a parientes o amigos en su casa.	Visit relatives or friends at home
	1	Sí	Yes
	2	No	No
1. Visit		En los últimos 7 días, ¿tú o alguien en tu hogar realizaron alguna de las siguientes actividades?	In the last 7 days, did you or someone in your household perform any of the following activities?
		Asistir a una reunión o fiesta con más de 10 personas	Attend a meeting or party with more than 10 people
	1	Sí	Yes
	2	No	No
1. Risky Inside		Ahora piensa en el riesgo de contagio. ¿Qué tan riesgoso crees que es ir a un gimnasio cerrado?	Now think about the risk of contagion. How risky do you think it is to go to an indoor gym?
	1	Riesgo alto	High risk
	0	Riesgo medio	Medium risk
	0	Riesgo bajo	Low risk
	.	No sé / prefiero no responder	I don't know / I prefer not to answer
1. Social Distance		Pensando en tus vecinos y conocidos, ¿dirías que en general toman o no toman las siguientes medidas? Mantener sana distancia de otras personas	Thinking about your neighbors and acquantances, would you say that in general they follow the following measure? Keep social distance from others
	1	Sí	Yes
	0	No	No
