# Supplementary material for: Do you have COVID-19? How to increase the use of diagnostic and contact tracing apps
Source: PLoS One. 2021 Jul 29;16(7):e0253490. doi: 10.1371/journal.pone.0253490 (PMC8321141; doi:10.1371/journal.pone.0253490)
Supplement: Attachment — Submitted filename: Response to Ref2_PLOSONE_R&R_Final.pdf [file pone.0253490.s011.pdf]

****

RESPONSES TO REVIEWER 2

****

We are happy to report that the attached revised version addresses all of the comments you raised. For your convenience, in what follows we include your comments in *italics* above our itemized responses in **bold**.

> *Reviewer #2: The paper presents original results about the way governments' messages regarding privacy safeguards, contrary to their intended aim, may actually trigger concerns and undermine citizens' willingness to download COVID-19 symptom checking and contact tracing apps. I have read the article with great interest. I think the paper is solid and well-written.*

We appreciate the comments made by the reviewer.

> *I have only one suggestion that I hope the authors will be willing to take into account. The discussion section could be expanded and engage with two bodies of literature. On the one hand the empirical literature on public attitudes towards COVID-19 apps in other geographical contexts. For instance:*
>
> *Saw, Y.E., Tan, E.Y.Q., Liu, J.S. and Liu, J.C., 2021. Predicting public uptake of digital contact tracing during the covid-19 pandemic: results from a nationwide survey in Singapore. Journal of medical Internet research, 23(2), p.e24730.*
>
> *Zimmermann, B.M., Fiske, A., Prainsack, B., Hangel, N., McLennan, S. and Buyx, A., 2021. Early perceptions of COVID-19 contact tracing apps in German-speaking countries: comparative mixed methods study. Journal of medical Internet research, 23(2), p.e25525.*

We thank the reviewer for these very useful recommendations. These references help us to present the current discussion in a broader context.

> *On the other the literature (mostly in the field of ethics) stressing privacy as the main (ethical) concern, especially in the case of contact tracing apps. For instance*

Mello, M.M. and Wang, C.J., 2020. Ethics and governance for digital disease surveillance. Science, 368(6494), pp.951-954.)

Parker, M.J., Fraser, C., Abeler-Dörner, L. and Bonsall, D., 2020. Ethics of instantaneous contact tracing using mobile phone apps in the control of the COVID-19 pandemic. Journal of Medical Ethics, 46(7), pp.427-431.

This literature focuses mostly on privacy and autonomy (voluntariness) in their ethical analysis.

Some other scholars in ethics instead have highlighted the narrow focus on privacy, see:

Martinez-Martin, N., Wieten, S., Magnus, D. and Cho, M.K., 2020. Digital contact tracing, privacy, and public health. Hastings Center Report, 50(3), pp.43-46.

It would be nice if you could situate your results in this broader context.

The ethical issues surrounding the deployment of the apps are of utmost importance. We have now included these citations in the text to provide a more complete framework for our discussion of the problem and results.

On last observation. Your point (e.g. in the last paragraph) that focusing on benefit increases the willingness to download the app, is a bit undersold. Discussing it in light of the above mentioned debates would do it more justice. But other than that, at a more general level, this kind of consideration reminded me of a - now dated - paper by bioethicist Bruce Jennings on Public Health and Civic Republicanism (available here: https://www.researchgate.net/publication/262724398_Public_Health_and_Civic_Republicanism). In this paper Jennings argues that exclusive emphasis on human rights and individual interest will not do the job of providing a credible public justification to public health measures (page 57). Jennings appeals instead to messages centred around civic virtues (i.e. doing it for the good of all). I thought you could take this into account as a theoretical backdrop towards the end of your discussion.

We appreciate this suggestion. We now acknowledge the importance of positive messages to increase civic virtue and trust in health policy, which is fundamental for its effectiveness.
