# Supplementary material for: Do you have COVID-19? How to increase the use of diagnostic and contact tracing apps
Source: PLoS One. 2021 Jul 29;16(7):e0253490. doi: 10.1371/journal.pone.0253490 (PMC8321141; doi:10.1371/journal.pone.0253490)
Supplement: Attachment — Submitted filename: PLoSOne_review_20210406.docx [file pone.0253490.s010.docx]

*Comments on “Do you have COVID-19? How to increase the use of diagnostic and contact tracing apps”*

This study investigates the important issue of uptake of COVID-19 related apps intended for self-diagnosis and digital contact tracing. Using a survey experiment among a large sample of Facebook users in Mexico, it asks whether emphasizing data privacy, as many governments have done, is a useful messaging strategy or whether it might backfire by making privacy concerns more salient to the public. The authors find evidence in support of this idea: survey respondents primed with a message about data privacy were less likely to state that they would download hypothetical diagnostic and contact tracing apps, compared to a control group and to groups that received other messages.

This is an important contribution as governments and app developers consider why the uptake of such apps has been low and the study is generally well-executed. However, I have several suggestions for improvement, mostly related to the interpretation of certain results, scope conditions, and interpretation and implications. I discuss each of these, listed roughly in order of importance, then list some additional, more minor comments.

**Interpretation of mechanisms analysis:** The interpretation of the analysis of mechanisms (section 3.1) is misleading as currently written. The authors imply that they estimate conditional effects of the priming treatment, writing on p. 4 "We provide evidence that priming could be the mechanism at work: those who agreed with the priming statement showed stronger effects than whose who disagreed" and on p. 12 "If concerns about data privacy really drove the estimated negative effect of T3 [the data privacy prime] on the likelihood of downloading the contact tracing app, one would expect the effects of T3 to be larger (more negative) among those who responded 'Yes' to the follow-up question than among those who responded 'No.'") However, the analysis implied here is not possible given this particular experimental design. More precisely, it is not possible to compare treatment *effects* among those who answered 'Yes' and those who answered 'No' to the data privacy question because this question is only asked of respondents in the T3 group and not of those in the control group. Instead, what they show is that individuals in the T3 group who said they were concerned about data privacy were less likely to say they would download the apps than those who said they were not. This is not particularly surprising and does not speak directly to the mechanisms explaining the treatment effects of the prime. The authors could instead consider using an instrumental variables approach to estimate a local average treatment effect for respondents who were induced by the prime to be concerned about data privacy, although certain needed assumptions (i.e. the exclusive restriction) may not be plausible in this case. Alternatively, if they were to repeat the study they could ask the question about data privacy *all* respondents (following the prime for treated respondents), then use this as a manipulation check to see if the priming treatment indeed increased concerns about data privacy.

**Scope conditions and external validity:** The authors should include a discussion of the conditions under which messages emphasizing data privacy are likely to backfire, to help readers consider external validity. Two potential scope conditions come to mind in particular. The first is the "baseline" salience of data privacy. Data privacy messaging may be less likely to discourage app uptake where data privacy is already a highly salient issue. Instead, in contexts where people are generally more concerned about data privacy, failure to address data privacy might reduce app uptake more than the priming mechanism the authors highlight. The authors might consider presenting cross-national survey data that situates Mexico in relation to other countries in terms of general concern about data privacy. The authors should also discuss how features of the sample might limit external validity along these lines even within Mexico: Facebook users who opt into an online survey AND who give explicit consent to have their responses used when asked (see p. 6) may be less concerned about data privacy than the general population (and thus the negative effect of priming data privacy on app uptake may be more pronounced among this group). One way the authors could probe this is by comparing experimental results and/or responses to the data privacy question among the sample recruited via Facebook with the sample recruiting via government email lists. The second potential scope condition involves trust in government. Both data privacy priming treatments mention that the Mexican government is working to protect data privacy. If citizens do not have trust in their government's willingness or ability to do this, they may find such statements concerning and this, rather than data privacy per se, may be driving the results (we might consider, for example, whether the findings would be the same if the treatment had emphasized the steps private app developers were taking to protect privacy). To understand how this may affect the interpretation, it might be helpful to show how Mexico compares to other countries in terms of "baseline" trust in government.

**Interpretation and implications:** The authors interpret their main findings to mean that any discussion of data privacy risks discouraging respondents from downloading COVID-19-related apps. However, they may wish to acknowledge that this outcome could depend on how privacy concerns are discussed. Both data privacy primes used in the experiments are quite vague: they raise the issue of data privacy without specifying *how* the government is addressing it, nor how it might be addressed in a particular app. Zhang et al (2020), for example, find that Americans were more likely to say they would download a hypothetical contact tracing app that stored data in a decentralized manner compared to an app that stored data centrally. Messaging that explains *how* privacy is protected in a particular app may be persuasive, even if general statements about data privacy are not.

**Minor comments**

- On p. 3 the authors cite a reference to a study stating that contact tracing apps require 80% uptake to be effective. I would point them to more recent statements by the authors who produced this study clarifying that these apps can begin to have a protective effect at much lower levels of uptake. See, for example: <https://www.technologyreview.com/2020/06/05/1002775/covid-apps-effective-at-less-than-60-percent-download>/
- On p. 7 the sentence "The first column of Table 1 provides basic descriptive statistics for the control group (these should be close to sample means due to the randomization of the treatment assignment)" is a bit strange. Pre-treatment covariate balance between experimental treatment groups on pre-treatment covariance is testable and, in fact, the authors do test this directly in Table 1.
- On p. 12, in the discussion of mechanisms, the authors assert that "the opposite pattern holds" for the other two treatment arms (T1 and T2). By this, they seem to mean that those who answered "yes" to the priming questions for T1 and T2 were more likely to state that they would download the apps compared to those who answered "no", while those who answered "no" to the priming question for T3 were more likely compared to those who answered "yes." It is not clear in what sense these patterns are opposite. In all three cases, people who gave the more "pro-online" or technology-friendly answer were more likely to say they would download a COVID-19-related app.
- In this same section, the authors write "The perceived convenience of, or satisfaction with online services in general are additional drivers of app adoption and overall a better strategy to motivate compliance with the policy." The aforementioned analysis does not support this. It shows that people who feel more favorably toward online services are more likely to say they would download COVID-19 apps, but this does not tell us anything about how to motivate people who are not already favorable toward both online services in general and toward COVID-19 apps specifically.
- The motivation for the first treatment arm regarding Facebook is slightly confusing and could use some additional explanation. Is the idea simply to prime the idea that mobile apps in general are useful?
- Was the study pre-registered? If so, the authors should specify this.
